# Composition and function of P bodies in *Arabidopsis thaliana*

**DOI:** 10.3389/fpls.2014.00201

**Published:** 2014-05-14

**Authors:** Luis D. Maldonado-Bonilla

**Affiliations:** Laboratory of Plant Molecular Biology, Instituto Potosino de Investigación Científica y Tecnológica, San Luis PotosíMexico

**Keywords:** P bodies, decapping, deadenylation, mRNA decay, post-transcriptional gene regulation, RNA-protein complex, stress

## Abstract

mRNA accumulation is tightly regulated by diverse molecular pathways. The identification and characterization of enzymes and regulatory proteins involved in controlling the fate of mRNA offers the possibility to broaden our understanding of posttranscriptional gene regulation. Processing bodies (P bodies, PB) are cytoplasmic protein complexes involved in degradation and translational arrest of mRNA. Composition and dynamics of these subcellular structures have been studied in animal systems, yeasts and in the model plant *Arabidopsis*. Their assembly implies the aggregation of specific factors related to decapping, deadenylation, and exoribonucleases that operate synchronously to regulate certain mRNA targets during development and adaptation to stress. Although the general function of PB along with the flow of genetic information is understood, several questions still remain open. This review summarizes data on the composition, potential molecular roles, and biological significance of PB and potentially related proteins in *Arabidopsis*.

## INTRODUCTION

The diversity of eukaryotic mRNA decay pathways illustrates the importance of nullifying transcripts as a mechanism to ensure proper course of gene expression and prevent the accumulation of aberrant mRNA (Houseley and Tollervey, 2009; Christie et al., 2011). These molecular mechanisms that abrogate accumulation of transcripts and proteins depend on the formation of RNA-protein complexes (RNPs). Under specific conditions or developmental cues, eukaryotic cells sort proteins, and mRNA in microscopically detectable complexes, commonly called cytoplasmic foci, categorized depending on the protein composition. Immunodetection of foci of the mice 5′-exoribonuclease XRN1 indicated that some pathways of RNA degradation might occur in protein speckles (Bashkirov et al., 1997). The 5′-exoribonuclease activity depends on the removal of 5′ cap structure 7-methyl-Guanosine-diphosphate (m7GDP). Interestingly, subsequent localization studies in yeast demonstrated that decapping-associated proteins are also localized in cytoplasmic foci, which were called Processing bodies, P bodies (PB; Sheth and Parker, 2003) or GW bodies in mammals (Eystathioy et al., 2003). Further microscopical and biochemical studies ended up proposing the composition of PB, which is conserved in eukaryotic organisms (Parker and Sheth, 2007). Similar structures have been identified in the protists *Trypanosoma cruzi* and *Entamoeba histolytica *(Holetz et al., 2007; López-Rosas et al., 2012). Electronic microscopy observations in human cells revealed that PB are clustered strands of 10–15 nm of diameter (Souquere et al., 2009).

Formation of PB depends on the availability of free cytoplasmic mRNA or mRNA not associated with polysomes (Teixeira et al., 2005). This correlation has been reported in *Arabidopsis* by trapping mRNA into polysomes by short treatments with cycloheximide (CHX; Weber et al., 2008). However, endogenous signaling events influence the formation of PB in *Saccharomyces cerevisiae *(Kilchert et al., 2010; Ramachandran et al., 2011).

The mRNAs aggregated into PB are translationally repressed and potentially degraded, even their re-incorporation in ribosomes is plausible. Decapping, deadenylation and 5′-exonucleolytic decay are biochemically related enzymatic modifications catalyzed in PB. Besides their involvement in RNA decay, these proteins may secure the translational arrest. Accumulation of related decay factors in PB implicates spatially ordered mechanisms of post-transcriptional regulation. As the activity of such factors in modifying mRNA is most likely irreversible, other mechanisms, possibly occurring outside PB and before the transcript is loaded into them, are necessary to accomplish a selective and controlled delivery, degradation, or translational arrest. It is crucial for the living cell because the knockout of some of those components causes lethality or severely abnormal phenotypes in *Arabidopsis*.

The intrinsic dynamics of PB in controlling the mRNA fate could lead a functional link between PB and other mRNA-containing cytoplasmic foci, for example, stress granules (sg) and heat shock granules (HSG). In human cells, SG are composed by proteins related to initiation of translation such as eukaryotic initiation factors (eIF3, eIF4E, eIFG), ribosomal subunits, and poly(a)-binding protein1 (PABP-1; Anderson and Kedersha, 2008). Assembly of SG is triggered by environmental stress and requires mRNA associated with 48S complexes, thus, assembly can be disrupted by CHX. The HSG described in *Arabidopsis* and tomato are formed upon prolonged heat treatment, they contain heat shock proteins and polyadenylated mRNA (Nover et al., 1989; Weber et al., 2008). The physical proximity between SG/HSG and PB has been evidenced in human cells and *Arabidopsis* (Kedersha et al., 2005; Weber et al., 2008). Such proximity might be necessary for the proper sorting of mRNAs after release form polysomes or to facilitate the exchange of mRNAs and proteins in a confined subcellular space. As SG formation is triggered by stress, they might protect transcripts during the lapse of stress and re-initiate translation once conditions get more favorable. If the translation of some transcripts aggregated into SG is not required, the proximity between foci might facilitate transport and decay into PB. Although some proteins related to RNA metabolism can associate with SG (Anderson and Kedersha, 2008), modification of mRNA targets into SG should be demonstrated. In contrast, the battery of proteins involved in RNA decay contained in PB favors a scenario where target mRNAs are translationally repressed and degraded, either they are released from polysomes, SG, or directly delivered after nuclear export.

## COMPOSITION OF P BODIES

**FIGURE 1:**
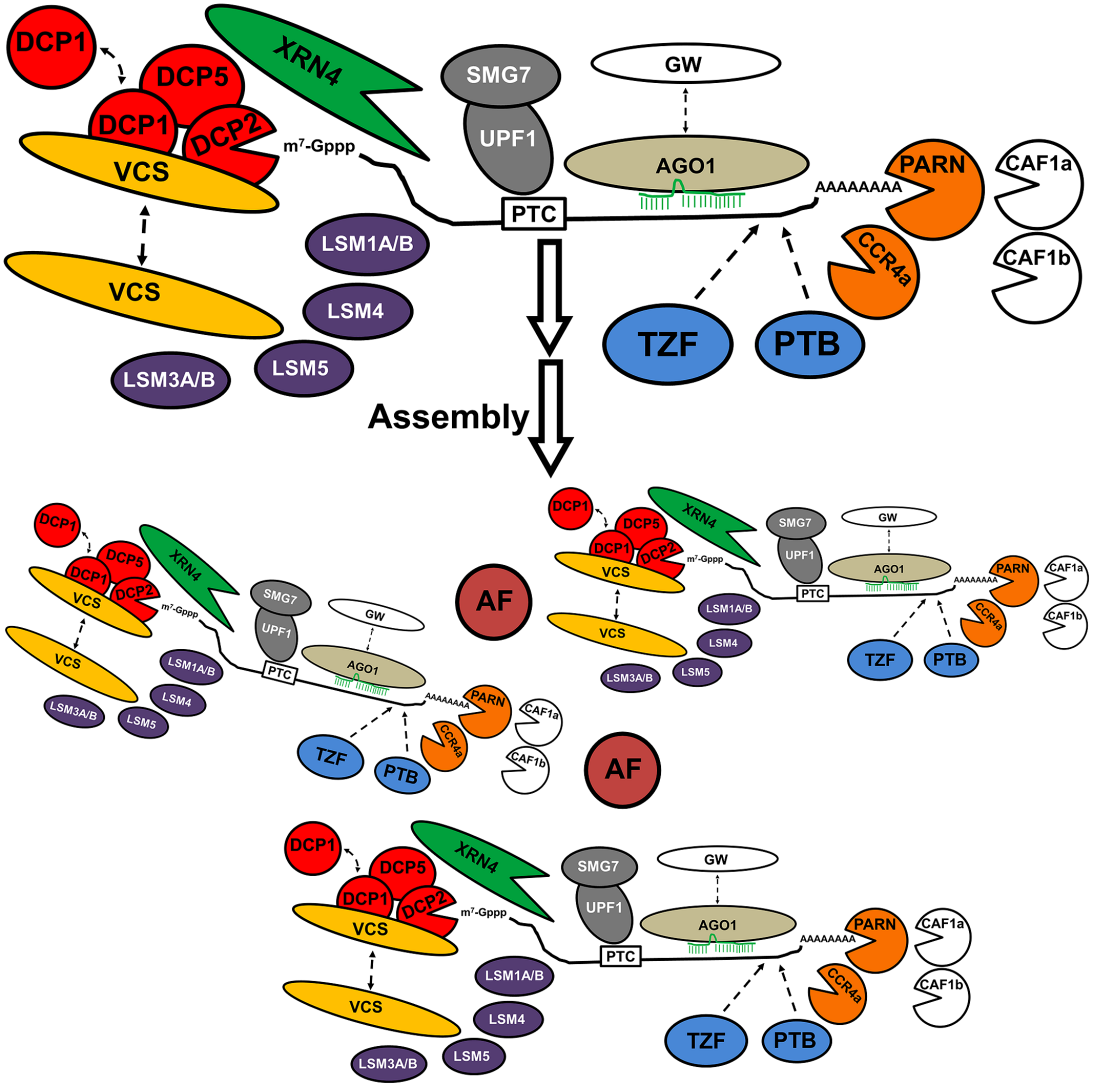
**Composition of P body in *Arabidopsis thaliana*.** Proteins involved in decapping (DCP1, DCP2, DCP5, VCS, LSM); deadenylases (PARN, CCR4a); the 5′-exoribonulease 4 (XRN4), microRNA-dependent endonuclease (AGO1); proteins of non-sense mediated RNA decay (UPF1, SMG7), and RNA-binding proteins such as tandem zinc fingers proteins (TZF) and polypyrimidine-binding proteins (PTB) have been detected in PB. Localization of the Deadenylases CAF1a, CAF1b, and GW proteins has to be experimentally confirmed. Unknown “Aggregation Factors” (AF), such as Q-rich proteins, might mediate the assembly of the structure, possibly by joining individual components or fusion of independent PB. The mRNA trapped into the PB is translationally arrested, and is eventually degraded by the decay factors, or return to polysomes to be translated.

Subunits of decapping complex, the 5′-exoribonuclease, and deadenylases are prominent markers of PB in eukaryotic organisms. Such core decay factors are distinguishable from other accessory proteins because they have the potential to modify the structure of mRNA. RNPs accessing into PB may be decorated with proteins that can interact with decay factors to mediate their activity within PB. Those proteins are distributed in different subcellular locations, including PB. Their frequency of association with PB might be synchronized with endogenous or exogenous signals that control the fate of specific mRNAs as a mechanism to fulfill requirements of the cell. The microRNA (miRNA)-dependent endonuclease argonaute1 (AGO1), components of nonsense-mediated mRNA decay (NMD) and RNA-binding proteins such as tandem zinc finger proteins occasionally localize in PB, as so far, their functional connection to PB is hypothetical in *Arabidopsis*. The **Figure 1** illustrates reported and putative PB components in *Arabidopsis*.

### DECAPPING COMPLEX

The cleavage of the 5′ cap compromises mRNA to 5′ to 3′ exonucleolytic decay, apparently in an irreversible way, hence, decapping activity is tightly regulated (Li and Kiledjian, 2010). In yeast and animals, decapping complex consists on several subunits such as DCP1, DHH1p, EDC3, SCD6, PAT1, and LSM1-7 which mediate the activity of the catalytic subunit DCP2 (Kulkarni et al., 2010; Zheng et al., 2011). The decapping complex in *Arabidopsis* is formed by the catalytic subunit DCP2, and the subunits DCP1, DCP5, and varicose (VCS; Xu et al., 2006), although other proteins can potentially affect decapping activity.

The *Arabidopsis* DCP2 harbors a conserved nudix hydrolase domain essential for the decapping activity, and physically interacts with DCP5, and VCS (Xu et al., 2006). In the same report. Xu et al. (2006) demonstrated that DCP1 stimulates decapping activity *in vivo*. DCP1 contains an EVH1-like domain potentially involved in protein-protein interactions (She et al., 2004). DCP1 interacts with itself, and experimental evidences in *Nicotiana benthamiana* demonstrated DCP1 does not interact with DCP2 (Iwasaki et al., 2007), indicating that additional components are required to link the function of DCP1 to the catalysis of DCP2. DCP1 and DCP2 may be physically close each other due to their interaction with VCS. Interaction between VCS, DCP1, and DCP2 was demonstrated *in vivo* while yeast two hybrid experiments revealed interaction between VCS and DCP2 and VCS with itself (Xu et al., 2006; Goeres et al., 2007). VCS contains two protein–protein interaction WD40 motifs, although they are not necessary to the interaction with DCP1 and DCP2. Deletion of WD40 repeats in *Ge-1*, the human homolog of *VCS*, did not compromised the localization into PB, then, WD40 repeats probably function in the recruitment of another mediators of decapping (Yu et al., 2005).

The *Arabidopsis* DCP5 is required for decapping and repression of translation *in vivo *(Xu and Chua, 2009). PB visualized by expressing *DCP1*-*CFP* are smaller in knockdown *dcp5-1* compared to Col-0. This comparison indicates DCP5-mediated assembly of PB is necessary for proper decapping. Impaired translational repression or decapping in *dcp5-1* might cause the release of some mRNA form PB, thus, smaller size of PB in *dcp5-1* might be a consequence of their decreased capacity to retain mRNA. Interaction between DCP5 and the RNA chaperone cold shock domain protein 3 AtCSP3 was reported (Kim et al., 2013) and possibly indicates that unwinding secondary structures of RNA might be necessary to facilitate decapping or inhibition of translation. The yeast DEAD box helicase DHH1p and homolog proteins in animals promote decapping and inhibit translation (Kulkarni et al., 2010). *Arabidopsis* lacks a homolog of DHH1p, but other proteins might replace its function through analogous processes.

Pat1 (protein-associated with topoisomerases) is conserved in different organisms, involved in activation of decapping and inhibition of translation (Coller and Parker, 2005; Scheller et al., 2007). In concordance with the formation of PB and inhibition of translation, nutrient deprivation causes nuclear accumulation of tRNAs in yeast. The yeast *dhh1*Δ and *pat1*Δ mutants failed to accumulate tRNAs into nucleus during starvation, but *xrn1*Δ showed normal tRNA nuclear accumulation (Hurto and Hopper, 2011). These evidences suggest that the formation of PB is accompanied by the nuclear accumulation of tRNAs in a DHH1p-Pat1-dependent manner as an mechanism to ensure inhibition of translation. The *Arabidopsis* genes *At1g79090*, *At3g22270*, and *At4g14990* are annotated as *Pat1* (http://www.arabidopsis.org), experimental evidences are required to confirm their localization and molecular function.

Sm-like proteins (LSM) interact each other to form complexes to activate decapping and modulate splicing in yeast (Tharun et al., 2000). The *Arabidopsis* LSM1-7 proteins form a cytoplasmic heptameric complex. LSM1A-GFP, LSM1B-GFP, LSM3A-GFP, LSM3B-GFP, and LSM4-GFP localize to cytoplasmic foci in root tips of stably transformed plants (Perea-Resa et al., 2012). Formation of these foci is sensitive to CHX, and the co-localization with RFP-DCP1 confirmed their physical association with PB. Heat stress facilitates the detection of LSM1-4-GFP containing cytoplasmic foci, it tempts to consider that LSM proteins are simultaneously incorporated in distinct cytoplasmic foci. Given that cytoplasmic foci localization of GFP-DCP2 and GFP-VCS is depleted in *lsm1alsm1b*, both proteins might participate in the assembly of PB or, at least influence the localization of VCS and DCP2.

Decapping activity is prevented by the translation initiation factor eIF4E, a 5′ cap-binding protein (von der Haar et al., 2004). Its localization in PB and SG is documented in humans and yeast (Andrei et al., 2005; Brengues and Parker, 2007), but the homolog of *Arabidopsis* was only detected in SG (Weber et al., 2008).

### 5′-EXORIBONUCLEASE

Decapped mRNA is prone to 5′ to 3′ decay. The *Arabidopsis XRN4 *complements the yeast* xrn1*Δ mutant (Olmedo et al., 2006). XRN4 co-localizes to DCP1 in *Arabidopsis*, furthermore, PB visualized with DCP1 antiserum were quite observable in the knockout *xrn4* line compared to wild type plants (Weber et al., 2008). This interesting comparison suggests the loss of 5′-exoribonuclease activity in *xrn4* enhances the assembly of PB by over accumulation of potentially uncapped mRNA. XRN4 might prevent accumulation of uncapped intermediates potentially involved in short interfering (siRNA) biogenesis (Gregory et al., 2008), and also participates in the degradation of selected 3′ end products from miRNA-mediated decay (Rymarquis et al., 2011).

### DEADENYLATION

Deadenylases and their regulatory proteins play an essential role both in 5′ to 3′ RNA decay and formation of PB (Zheng et al., 2008). Polyadenylated mRNA is aggregated in SG and HSG of tomato cells, thus, deadenylation might be necessary to sort mRNAs into different foci. Shortening of poly-A might exclude poly-A binding proteins and proteins associated with translation to decrease the efficiency of translation and produce mRNAs accessible to PB components. The deadenylation is catalyzed by the poly-A-specific ribonuclease AtPARN and the carbon catabolite repressor 4-CCR4 associated factor 1-CAF1 complex (CCR4–CAF1). Both AtPARN and CCR4a co-localize to DCP1 in *Agro*-infiltrated tobacco leaves (Moreno et al., 2013). The activity of CCR4–CAF1 complex is regulated by the NOT1-5 proteins in yeast and humans, nevertheless their function has not been confirmed in *Arabidopsis* (Zheng et al., 2011; Abbasi et al., 2013).

### ARGONAUTE1

ARGONAUTE (AGO) proteins have miRNA-dependent RNA slicer activity and are part of the RNA interference effector complex (RISC). Interaction between AGO proteins and the PB-associated GW182 (TNRC6A) is critical to gene silencing in humans (Lazzaretti et al., 2009). Early immunofluorescence studies of GW182 (TNRC6A) in mammalian cells revealed co-localization with proteins of decapping in foci, were initially known as GW-bodies (Eystathioy et al., 2003). Human AGO1 and AGO2 co-localize to DCP1 (Liu et al., 2005). GW182 and its isoforms contain a GW motif and a Q-rich region which contributes to the localization into PB, but is not required for silencing (Eulalio et al., 2007). Q or N-rich regions in other PB-related proteins might provoke a general aggregation of other components (Reijns et al., 2008). GW-containing proteins have been identified *Arabidopsis* by a computational approach. However, experimental evidence is required to confirm the potential association with AGO proteins (Karlowski et al., 2010; Gu et al., 2012). *Arabidopsis* AGO1 is associated with microRNAs, endogenous, and transgene derived siRNAs (Baumberger and Baulcombe, 2005), its potential localization in PB is documented (Pomeranz et al., 2010a). The accumulation of 3′-end products of miRNA-mediated decay in the *Arabidopsis xrn4* mutant suggests that AGO slicer activity acts upstream XRN4, possibly associated with PB (Rymarquis et al., 2011). Moreover, the increase of potential targets of miRNA in *dcp1*, *dcp2,* and *vcs* suggests a functional link between decapping and AGO1 activity (Motomura et al., 2012).

Localization of AGO1 varies under different conditions. *GFP-AGO1 *driven by the native* AGO1 *promoter is detectable in cytosol, nuclear envelope and the proximity to Golgi apparatus (Derrien et al., 2012). The viral suppressor P0 triggers the localization of GFP1-AGO1 into vesicles as a necessary early step in its degradation by autophagy.

The translational repression activity of AGO1 has been uncovered for specific miRNA targets. For example, the superoxide dismutase *CSD2* mRNA is targeted by the miR398 to repress translation under “low Cu^+^^+^” conditions, this mechanism requires VCS (Brodersen et al., 2008). Both miRNA and AGO1 are detectable in polysomes to repress translation of the targets independent of the AGO1 slicer activity (Lanet et al., 2009). These results proposed that AGO1 and VCS sequester mRNA into PB to prevent their translation. Nevertheless, the translational repression activity of AGO1 rather requires the integral Endoplasmic reticulum (ER) protein altered meristem program1 (ATM1) and possibly its paralog LAMP1 (Li et al., 2013). Increased amounts of miRNA target transcripts such as *AGO1, PHB, *and* CSD2 *in membrane bound polysomes of the double mutant* atm1lamp1 *suggests that ATM1 and LAMP1 displace miRNA target transcripts from translation in membrane bound polysomes. The specificity in the inhibition might depend in the miRNA-dependent ability of AGO1 to recognize unique mRNAs. Although VCS participates in translational arrest, the evidences illustrate that the AGO1-dependent translational inhibition is related to ER and not to PB.

### NONSENSE-MEDIATED mRNA DECAY

The NMD refers a surveillance system that detects and eliminates mRNAs containing premature termination codons (PTC) and also controls expression of wild type genes with long 3′-UTR, mRNAs harboring 3′-UTR-located introns, and upstream open reading frames (uORF)-containing genes, defined as small ORFs encoded in the 5′-UTR of major ORFs (mORF; Hayden and Jorgensen, 2007; Kerényi et al., 2008). The up-frameshift proteins UPF1, UPF2, and UPF3 are essential factors of NMD. In yeast, UPF1-3 are involved in recognition and targeting PTC-containing mRNA to PB (Sheth and Parker, 2006; Nyikó et al., 2009). UPF1-3 are cytoplasmic, but they co-localize to DCP2 in the *dcp1*Δ strain, theoretically by accumulation of non-degraded aberrant mRNAs or other substrates of NMD.

*Arabidopsis* UPF1 is also cytoplasmic, and its incorporation in PB depends on 14-3-3-like protein SMG7 (Mérai et al., 2012). A RNP formed by the NMD target, the presumably phosphorylated UPF1, UPF2, and UPF3 could be transported to PB by SMG7 to promote the degradation of the target. As several components of NMD -such as* SMG5* and *SMG6*- are absent in *Arabidopsis*, the localization of UPF1 and SMG7 in PB would indicate that 5′ to 3′ decay could have a predominant role in NMD. *XRN4*-silenced tobacco leaves accumulated PTC-containing mRNA at equal levels than the control leaves (Mérai et al., 2012). This results contradicts the model in yeast, where XRN1 is involved in NMD. So far, the role of decapping complex in plant NMD is not demonstrated, and evidences of 3′ to 5′ decay in PB are lacking. If decapping is dispensable for plant NMD, XRN4 would be dispensable as well. Plant XRN4-independent events of NMD and the role of decapping in this pathway have to be clarified in the future.

### TANDEM ZINC FINGER PROTEINS AND OTHER RNA-BINDING PROTEINS

RNPs shuttle between cytoplasm-polysomes-PB and other cytoplasmic foci as a dynamic mechanism to regulate the fate of mRNAs. Microscopical observations and analysis of polysomal profiles revealed movement of mRNA to PB triggered under glucose deprivation in yeast; and selected mRNAs are newly detectable in polysomal fractions after readdition of glucose (Brengues et al., 2005). The mRNAs harbor diverse *cis*-elements potentially recognized by RNA-binding proteins, siRNAs or microRNAs that regulate their maturation, stability, translation rate, as well as localization elements or Zipcodes, involved in the delivery of transcripts to specific subcellular compartments (Ahmed et al., 2011). Specificity in the reactions catalyzed in PB might be conferred by RNA-binding proteins involved in the recognition of targets prior to their delivery in PB.

The human tandem CCCH-type zinc finger proteins such as TTP, are detectable in PB. TTP exists in complex with the proteins DCP1, DCP2, and XRN1 (Lykke-Andersen and Wagner, 2005; Franks and Lykke-Andersen, 2007). The RNA-binding activity of TTP and similar proteins is conferred by the CCCH-type zinc finger motifs. TTP binds to the class II AU-rich sequence 5′-UUAUUUAUU-3′ (class II ARE) located in the 3′-UTR of specific mRNAs. TTP enhances decapping of ARE-containing RNA *in vitro, *which suggests that targets of TTP are prone to decapping after their delivery into PB (Fenger-Grøn et al., 2005). Beyond its role in ARE-containing mRNA decay, TTP has been postulated as a nucleation factor of PB since microscopic detection of GFP-HsDCP1 increases by overexpression (OE) of *TTP *(Franks and Lykke-Andersen, 2007).

The *Arabidopsis* CCCH-containing Tandem Zinc Finger protein family consists of 11 members. Transient expression in protoplasts revealed all of them localize to cytoplasmic foci, and the co-localization with PB and SG markers has been demonstrated for specific members (Pomeranz et al., 2010a,b; Bogamuwa and Jang, 2013; Maldonado-Bonilla et al., 2014).

Localization of TZF proteins is highly dependent on the experimental conditions that influence the physiology and requirements of the cell. Nuclear, cytoplasmic, and nucleocytoplasmic distribution was reported to SZF1/TZF11, SZF2/TZF10, OXS2/TZF7, TZF2, and TZF3, by using either transient expression in onion epidermal cells or stable expression in transgenic plants (Sun et al., 2007; Blanvillain et al., 2011; Lee et al., 2012). Those reports ruled out any localization in cytoplasmic foci or PB.

*In planta* detection of TZF1 in putative PB depends on Methyl Jasmonate (MeJA) treatment or wounding (Pomeranz et al., 2010a) unlike TZF4, TZF5, and TZF6, whose localization in putative PB is detectable in intact plants (Bogamuwa and Jang, 2013). TZF1 accumulates into the nucleus by inhibiting XPO1-mediated nuclear export with Leptomycin B (LMB; Pomeranz et al., 2010a), evidencing a nucleocytoplasic shuttling. Once in cytoplasm, TZF1 has potential to be incorporated in PB or SG, though the signals, factors or functional domains of TZF1 that promote assembly in PB are still unknown. The localization of TZF9 is also affected by LMB in *Arabidopsis* protoplasts, however, further experiments has to be performed in order to confirm the nucleocytoplasmic shuttling (Maldonado-Bonilla et al., 2014). The nucleocytoplasmic shuttling of TZF proteins might indicate they have different functions both in nucleus and cytoplasm, or perhaps, that the proteins recognize the potential mRNA target during or after transcription. A hypothetical RNP formed in the nucleus might determine the fate of the mRNA once exported in the cytoplasm.

*In vitro* evidences indicate that TZF proteins bind to RNA, DNA, or even possess ribonuclease activity (Pomeranz et al., 2010a; Blanvillain et al., 2011; Lee et al., 2012; Maldonado-Bonilla et al., 2014). Directs mRNA targets of *Arabidopsis* TZF proteins have not been reported so far, and critical amino acid substitutions in the zinc finger motifs might entail their inability to recognize the class II ARE sequence. However, the arginine-rich region preceding CCCH zinc fingers also contributes in the RNA-binding activity (Qu et al., 2014).

*Arabidopsis* polypyrimidine tract-binding proteins PTB1, PTB2, and PTB3 are involved in alternative splicing -a nucleus-localized process-, however they are also detectable in PB (Stauffer et al., 2010). As alternative splicing can produce PTC-containing mRNAs (Wang and Brendel, 2006), these proteins might traffic to PB as part of the mechanism of degradation of substrates of NMD.

The human poly-C binding protein 2 (PCBP2) shuttles between nucleus and cytoplasm, and is detectable in PB and SG. Different RNA-binding KH domains are critical to confer its localization in both cytoplasmic foci (Fujimura et al., 2008). This changing localization may be necessary to join different protein complexes to regulate translation or transcript stability. *Arabidopsis* possesses 26 KH-containing proteins, the arrangement and architecture of KH domains of PEPPER (At4g26000) and At1g14170 are reminiscent to PCBP2 (Lorkoviæ and Barta, 2002; Ripoll et al., 2006). Subcellular localization studies on these proteins might complement their characterization.

The subcellular dynamics of RNA-binding proteins mentioned above might be related to direct or indirect interaction with cytoskeletal proteins. Myosin Va co-localizes to PB markers, co-precipitates with the 5′cap-binding protein eIF4E, and is required to the assembly of PB in HeLa cells (Lindsay and McCaffrey, 2011). Although eIF4E is not accumulated in PB in plants, direct interaction between myosins and DCP1 has been recently reported in *Arabidopsis* (Steffens et al., 2014). This evidence might motivate to further investigate movement of PB and RNP along actin filaments or microtubules.

## BIOLOGICAL RELEVANCE OF PB COMPONENTS IN DEVELOPMENT AND RESPONSE TO STRESS

Analysis of the phenotype of mutant lines has uncovered the importance of PB machinery for plant development and responses to stress. The **Table 1** summarizes *Arabidopsis* PB-related genes and the phenotypes of the corresponding knockout and OE lines.

**Table 1:** Knock-out and over expression (OE) of P body-related genes alters development and adaptation to stress (view text for details).

Gene (AGI code)	Function	Mutant/OE line	Phenotype	Reference
**Decapping**
*DCP1 (At1g0837)*	Regulatory subunit	*dcp1-1, dcp1-2*	Post-embryonic lethal	Xu et al. (2006), Iwasaki et al. (2007)
*DCP2/TDT (At5g13570)*	Catalytic subunit	*dcp2-1, tdt-1*	Post-embryonic lethal, severe morphological defects	Xu et al. (2006), Goeres et al. (2007)
*DCP5 (At1g26110)*	Regulatory subunit	*dcp5-1*	Pale and weak cotyledons, abnormal rosette leaves, and vascular patterning	Xu and Chua (2009)
*VCS (At3g13300)*	Subunit of decapping complex, scaffold	*vcs-1, vcs-7*	Abnormal leaves, vascular defects, temperature-sensitive	Goeres et al. (2007), Xu and Chua (2009)
*LSM1a (At1g19120)*	Promoter of decapping, assembly of PB	*lsm1alsm1b *(double knockout)	Growth delay, abnormal leaves, lack of apical dominance	Perea-Resa et al. (2012), Golisz et al. (2013)
*LSM1b (At3g14080)*	Promoter of decapping, assembly of PB	*lsm1alsm1b *(double knockout)	Growth delay, abnormal leaves, lack of apical dominance	Golisz et al. (2013)
*LSM5/SAD1 (At5g48870)*	Promoter of decapping and splicing	*sad1*	Enhanced ABA-sensitivity	Xiong et al. (2001)
**5′-Exoribonuclease**
*XRN4/EIN5 (At1g54490)*	5′-exoribonuclease	*ein5-1, ein5-6, xrn4-3*	Ethylene-insensitive	Olmedo et al. (2006), Potuschak et al. (2006)
**Deadenylation**
*PARN (At1g55870)*	Poly-rA-ribonuclease	*parn-1, parn-2*	Embryogenesis arrest	Reverdatto et al. (2004)
*CAF1a (At3g44260)*	3′-5′ Deadenylase	*Atcaf1a-1*	Enhanced susceptibility to *Pseudomonas syringae. *Altered response to ROS and NaCl	Liang et al. (2009), Walley et al. (2010)
		*35S::CAF1a*	Increased response to *P. syringae*	Walley et al. (2010)
*CAF1b (At5g22250)*	3′-5′ Deadenylase	*Atcaf1b-2*	Altered response to ROS and NaCl	Walley et al. (2010)
**RNA-induced silencing**
*AGO1 (At1g48410)*	microRNA-dependent endonuclease.	*ago1-24, ago1-25, ago1-26*	Dark green, and serrated leaves. Self-sterility	Morel et al. (2002)
	Translational repressor.	*ago1-27*	Hyper susceptible to cucumber mosaic virus	
**Nonsense-mediated mRNA decay**
*UPF1 (At5g47010)*	Component of NMD	*upf1-3*	Post-embryonic lethal	Arciga-Reyes et al. (2006)
		*upf1-5*	Dwarf, curly leaves. Increased resistance to *P. syringae*	Jeong et al. (2011)
*SMG7 (At5g19400)*	Component of NMD, 14-3-3-like	*smg7-1, smg7-2, smg7-3*	Dwarf, constitutive defense responses, infertility	Riehs-Kearnan et al. (2012)
**Tandem zinc finger proteins**
*TZF1 (At2g25900)*	RNA/DNA binding	*35S:TZF1*	Enhanced sensitivity to ABA and tolerance to abiotic stress	Lin et al. (2011)
*TZF2/OZF1 (At2g19810)*	Ribonuclease activity (*in vitro*)	*35S:OZF1*	Enhanced tolerance to oxidative stress	Huang et al. (2011)
		*35S:TZF2*	Enhanced sensitivity to ABA and decressed response to JA	Lee et al. (2012)
*TZF3 (At4g29190)*	Ribonuclease activity (*in vitro*)	*35S:TZF3*	Enhanced sensitivity to ABA and decreased response to JA	Lee et al. (2012)
*TZF4 (At1g03790)*	?	*som-1, som-2, som-3, tzf4*	Early germination, irrespective of light regimen	Kim et al. (2008), Bogamuwa and Jang (2013)
		*35S:TZF4*	Delayed germination, dark green and smaller size	Bogamuwa and Jang (2013)
*TZF5 (At5g44260)*	?	*tzf5*	Early germination	Bogamuwa and Jang (2013)
		*35S:TZF5*	Delayed germination, dark green leaves, and smaller plant size	
*TZF6/PEI1 (At5g07500)*	?	*35S*:antisense*PEI1*	Abnormal embryo development, white seeds	Li and Thomas (1998), Bogamuwa and Jang (2013)
		*tzf6*	Early germination	
		*35S:TZF6*	Delayed germination, dark green leaves, and smaller plant size	
*TZF7 /OXS2 (At2g41900)*	Transcription factor	*oxs2-1, oxs2-2, oxs2-4, oxs2-6*	Early flowering, sensitive to diamide	Blanvillain et al. (2011)
		*35S:OXS2* (cytoplasmic version)	Delayed flowering	
*TZF9 (At5g58620)*	RNA-binding activity (*in vitro*)	*tzf9*	Attenuated response to pathogen-associated molecular patterns	Maldonado-Bonilla et al. (2014)
			Abnormal development of vascular tissue	Gandotra et al. (2013)
*TZF10 (At2g40140)*	?	*tzf10/szf2*	Increased susceptibility to *Botrytis cinerea *enhanced sensitivity to ABA and NaCl	AbuQamar et al. (2006), Sun et al. (2007)
		*35S:TZF10*	Tolerant to NaCl	Sun et al. (2007)
*TZF11 (At3g55980)*	?	*tzf11/szf1*	Enhanced sensitivity to ABA	Sun et al. (2007)
		*35S:TZF11*	Tolerant to NaCl	Sun et al. (2007)

Post-embryonic lethality of *Arabidopsis dcp1* and *dcp2 *mutants, and severe phenotypic defects in *vcs *and *dcp5 *mutants such as abnormal patterning of vascular tissue, defects in the shoot apical meristem, chlorosis, slow germination, and temperature sensitivity illustrate functional decapping components are essential for optimal plant development (Xu et al., 2006; Goeres et al., 2007; Iwasaki et al., 2007; Xu and Chua, 2009). Enhanced decapping activity is also necessary to adaptation to abiotic stress. *Arabidopsis sad1*(*lsm5*) is hypersensitive to ABA and drought (Xiong et al., 2001). *Arabidopsis* DCP1 is phosphorylated by the stress-related MAP kinase MPK6 in response to dehydration. Comparison of capped to uncapped transcripts ratios, and global transcript profiling in different mutants and transgenic lines unveiled that MPK6-mediated phosphorylation of DCP1 increases decapping activity in concert with DCP5 (Xu and Chua, 2012). As MAP kinase activity is triggered by other signals, it is worth to investigate whether decapping activity is modulated *via* phosphorylation of DCP1 in response to other adverse conditions.

The function of LSM proteins is crucial for plant growth (Perea-Resa et al., 2012). In *Arabidopsis*, some transcripts with prolonged half-life in *xrn4 *mutant are also stabilized in *lsm1alsm1b *and *lsm5 *mutants. Since capping is increased in some of the stabilized transcripts, LSM1A, LSM1B, and LSM5 might stimulate decapping (Golisz et al., 2013). The stabilization of mRNAs such as *WRKY40*, *WRKY33,* and* ACS6* in *lsm1alsm1b* and *lsm5* could be considered as evidence that modulation of decapping activity takes part during execution of plant defense to pathogens or response to stress (Golisz et al., 2013).

Disruption of *XRN4*/*EIN5* in *Arabidopsis* affects the response to ethylene. Reduction in the accumulation of the transcription factor EIN3 in *ein5* mutant is consequence of the increased levels of F-box proteins EBF1 and EBF2 which target EIN3 to degradation in an Ubiquitin-dependent manner (Olmedo et al., 2006; Potuschak et al., 2006).

Knockout of the deadenylase *AtPARN*, caused embryogenesis arrest, indicating this enzyme might be indispensable to deadenylate bulk mRNA during embryo development (Reverdatto et al., 2004). Selective deadenylation mediated by CCR4-CAF1 proteins is necessary to regulate responses to stress. Knockout mutant lines *atcaf1a* and *atcaf1b* display enhanced susceptibility to *Pseduomonas syringae *and altered response to abiotic stress (Liang et al., 2009; Walley et al., 2010). AtCAF1A and AtCAF1B are necessary to respond to *P. syringae*, possibly by deadenylating jasmonic acid-responsive mRNAs or negative regulators of plant defense. The higher sensitivity to reactive oxygen species (ROS) of *atcaf1a* and *atcaf1b *could be helpful to investigate the potential link between AtCAF1A/B, the response to ROS and the defense against *P. syringae.*

Severe vegetative and reproductive defects in *ago1* mutants confirms the importance of AGO1 for plant development. Hypomorphic *ago1* plants permitted higher accumulation of Cucumber Mosaic Virus (CMV) and displayed enhanced symptoms, which evidences the role of AGO1 in posttranscriptional gene silencing, virus resistance and degradation of exogenous RNAs (Morel et al., 2002).

### POTENTIAL FUNCTIONAL LINK BETWEEN NMD PATHWAY AND PB

NMD pathway is essential for seed development. Homozygous *upf1-2* and *smg7* are lethal (Yoine et al., 2006; Riehs-Kearnan et al., 2012). Weak mutant allele of *UPF1* (*upf1-5*) and *upf3* showed anomalies in vegetative growth and flowering (Arciga-Reyes et al., 2006). Elevated defense-related gene expression and salicylic acid (SA) accumulation of *upf3* may reflect the reduction in the growth of *P. syringae *(Jeong et al., 2011)*. *A selected group of *WRKYs *is up-regulated in *upf1-5* and *upf3*, suggesting UPF1/3 are involved in the decay of WRKY transcripts. Coincidently, CHX promotes accumulation of those WRKY mRNAs in wild type plants. The effect of CHX might imply the arrest of mRNAs in polysomes that impedes the recognition of UPF1 as an early step in NMD. XRN4 is dispensable for decay of PTC-containing transcripts (Mérai et al., 2012). Hence, targets of NMD could be transiently delivered within PB by UPF1 and SMG7 as a mechanism to inhibit translation, and, in some point, the target might be released from PB and degraded by other nucleolytic pathways. The link between UPF1 and the transport of mRNA targets to PB should be investigated in the future.

Conserved uORF homology groups have been identified in *Arabidopsis* and rice by following a *in silico* approach (Hayden and Jorgensen, 2007). Some genes encoding transcription factors, kinases, or enzymes of polyamine metabolism harbor uORF that potentially induces ribosome stalling or reduces stability of the mORF. If the translation or accumulation of uORF perturbates the translation of the mORF, the latter one gets prone to be recognized by components of the NMD pathway. Depletion of *GFP* expression driven by uORF in *Agro*-infiltrated *Nicotiana benthamiana* strengthen this potential mechanism, although not all the uORF-containing transcripts would be substrates of NMD (Nyikó et al., 2009). It is worth to investigate whether the decay of mORF depends on decapping, deadenylation, and 5′-exoribonuclease activity.

### IS THE FUNCTION TZF PROTEINS ASSOCIATED WITH PB?

Knockout and OE of *TZF* genes has revealed their involvement in ABA signaling and response to stress (AbuQamar et al., 2006; Sun et al., 2007; Blanvillain et al., 2011; Huang et al., 2011; Lin et al., 2011; Lee et al., 2012). The changing localization and *in vitro* RNA-binding and ribonuclease activity of TZF proteins suggests that their occasional association with PB might be biologically relevant. However, there are not evidences that indicate the phenotype of such mutants and OE lines is a consequence of mis-regulation of transcripts aggregated in PB.

The human CCCH protein TTP promotes mRNA decay by interaction with DCP2. Simultaneous presence of the target and TTP in PB has been demonstrated (Fenger-Grøn et al., 2005; Franks and Lykke-Andersen, 2007). The rice TZF1–GFP is detectable in PB upon salt stress, the recombinant protein binds to the 3′-UTR of selected stress related genes which are down-regulated in the OE line (Jan et al., 2013), indicating that OsTZF1 might lead the decay of these mRNA targets. Additional experiments have to be designed to investigate whether OsTZF1 destabilizes targets by stimulation of decapping. If this hypothesis is validated, similar experiments could be performed in *Arabidopsis* as starting point to demonstrate the functional link between TZF proteins and the decapping activity localized in PB.

## CONCLUDING REMARKS AND PERSPECTIVES

The aggregation of related decay factor in PB enables an organized set of mechanisms to prevent translation and catalyze the decay of specific transcripts. The core of plant PB is consensually known and different shuttling proteins might be involved in delivering mRNA targets and promoting degradation or storage via their interactions with core components, which is critical in regulating plant development and response to stress.

Current availability of genomes and transcriptome data encourages us to use the well-known information of *Arabidopsis* to *in silico* identify genes encoding components of PB as a first approach to study these foci in other plant models. The *in silico* analysis also allows the identification of *cis*-elements in potential mRNA targets.

Several methodologies to investigate RNA-protein interactions, isolation of polysomal mRNA and high resolution cell imaging used in yeast and animal systems have to be set up in model plants. Such technical package is necessary to further molecular characterization of proteins associated to PB, as well as the identification mRNA targets and RNA sequences recognized by RNA binding proteins. By overcoming technical challenges, we will broaden our understanding about the dynamics, plasticity, and mechanisms related to the PB function.

Finally, as knockout or overexpression of some PB-related proteins alters the response to environmental stresses, we can use those genes to design novel strategies of post-transcriptional gene regulation to improve crop protection and production.

## Conflict of Interest Statement

The author declares that the research was conducted in the absence of any commercial or financial relationships that could be construed as a potential conflict of interest.
